# Genetic variation and population structure of maize inbred lines adapted to the mid-altitude sub-humid maize agro-ecology of Ethiopia using single nucleotide polymorphic (SNP) markers

**DOI:** 10.1186/s12864-017-4173-9

**Published:** 2017-10-12

**Authors:** Berhanu Tadesse Ertiro, Kassa Semagn, Biswanath Das, Michael Olsen, Maryke Labuschagne, Mosisa Worku, Dagne Wegary, Girum Azmach, Veronica Ogugo, Tolera Keno, Beyene Abebe, Temesgen Chibsa, Abebe Menkir

**Affiliations:** 1 Bako National Maize Research Center, Ethiopian Institute of Agricultural Research (EIAR), Bako, West Shoa, Oromia, Ethiopia; 2 Department of Agricultural, Food and Nutritional Science, University of Alberta, 4-10 Agriculture/Forestry Centre, Edmonton, AB T6G 2P5 Canada; 3 International Maize and Wheat Improvement Center (CIMMYT), P. O. Box 1041, Village Market, Nairobi, 00621 Kenya; 4 Department of Plant Sciences, University of Free State, Bloemfontein, South Africa; 5 International Maize and Wheat Improvement Center (CIMMYT), P. O. Box 5689, Addis Ababa, Ethiopia; 6 International Institute of Tropical Agriculture, Oya road PMB, Ibadan, 5320 Nigeria

**Keywords:** Distance, GBS, Genetic purity, Heterogeneity, Heterotic grouping, Kinship, Population structure

## Abstract

**Background:**

Molecular characterization is important for efficient utilization of germplasm and development of improved varieties. In the present study, we investigated the genetic purity, relatedness and population structure of 265 maize inbred lines from the Ethiopian Institute of Agricultural Research (EIAR), the International Maize and Wheat Improvement Centre (CIMMYT) and the International Institute of Tropical Agriculture (IITA) using 220,878 single nucleotide polymorphic (SNP) markers obtained using genotyping by sequencing (GBS).

**Results:**

Only 22% of the inbred lines were considered pure with <5% heterogeneity, while the remaining 78% of the inbred lines had a heterogeneity ranging from 5.1 to 31.5%. Pairwise genetic distances among the 265 inbred lines varied from 0.011 to 0.345, with 89% of the pairs falling between 0.301 and 0.345. Only <1% of the pairs had a genetic distance lower than 0.200, which included 14 pairs of sister lines that were nearly identical. Relative kinship analysis showed that the kinship coefficients for 59% of the pairs of lines was close to zero, which agrees with the genetic distance estimates. Principal coordinate analysis, discriminant analysis of principal components (DAPC) and the model-based population structure analysis consistently suggested the presence of three groups, which generally agreed with pedigree information (genetic background). Although not distinct enough, the SNP markers showed some level of separation between the two CIMMYT heterotic groups A and B established based on pedigree and combining ability information.

**Conclusions:**

The high level of heterogeneity detected in most of the inbred lines suggested the requirement for purification or further inbreeding except those deliberately maintained at early inbreeding level. The genetic distance and relative kinship analysis clearly indicated the uniqueness of most of the inbred lines in the maize germplasm available for breeders in the mid-altitude maize breeding program of Ethiopia. Results from the present study facilitate the maize breeding work in Ethiopia and germplasm exchange among breeding programs in Africa. We suggest the incorporation of high density molecular marker information in future heterotic group assignments.

**Electronic supplementary material:**

The online version of this article (10.1186/s12864-017-4173-9) contains supplementary material, which is available to authorized users.

## Background

Maize (*Zea mays* L.) is the second most popular staple crop in Ethiopia after tef (*Eragrostis tef*) and its production in the country has doubled in less than two decades [1] . Currently, the total annual production and productivity has reached 7.2 million tons and 3.4 t ha^−1^, respectively (http://faostat3.fao.org), which is the second highest national average yield reported in sub Saharan Africa (SSA), only after South Africa [1]. Improved hybrids and open pollinated varieties (OPVs) developed by the national maize breeding program, in conjunction with introduced hybrids by multi-national seed companies, have significantly contributed to the rapid increase in maize production in the country [2]. However, maize productivity still remains far below the potential due to several factors, including inadequate farmers’ access to affordable quality seeds and mineral fertilizers, periodic drought, high incidence of pests and diseases, parasitic weeds, poor soil fertility, and scarcity of irrigation infrastructure. Genetic improvement of maize provides an option to address some of these constraints, but largely depends on the availability of genetic diversity, systematic classification and efficient utilization of the available germplasm.

Maize breeding program in Ethiopia started in early 1950’s focusing on evaluation and recommendation of OPVs for production [3]. Considering the narrow genetic base of the local maize germplasm at that time, the breeding program introduced temperate maize germplasm from USA, Europe and Israel to broaden the genetic base of maize germplasm in the country. However, temperate germplasm was not adapted to the growing conditions in Ethiopia. Ethiopia’s subsequent participation in the "East African Cooperative Maize Variety Trial" in the late 1960’s and early 1970’s enabled the identification of high yielding composites and hybrid varieties that were better adapted to the local growing conditions than those acquired in the 1950s [3], which was mainly due to agro-ecological similarity. In the 1980’s, the national breeding program started to introduce tropical maize germplasm from CIMMYT, IITA and other national programs in eastern Africa [3]. The introduction and evaluation of a wide range of maize germplasm over the years has enabled the national maize breeding program to develop and release several OPVs and hybrids for commercial production.

Breeders require detailed knowledge of inbred lines in order to (i) define core subsets of germplasm for specific traits [4], (ii) select parental combinations for developing progenies with maximum genetic variability for further selection [5] and (ii) describe heterotic groups [6–9]. A heterotic group is a collection of related inbred lines which tend to produce vigorous hybrids when crossed with lines from a different group, but not when crossed to other lines of the same group [10]. Maize breeders use handful of phenotypic traits for evaluating maize germplasm. Combining ability studies, mainly based on grain yield, is commonly used to assign inbred lines into distinct heterotic groups [3, 11]. Expression of phenotypic traits, however are often influenced by environmental factors which may affect the consistency and reliability of combing ability based classification. Therefore, use of molecular markers to characterize locally available inbred lines can complement and fine-tune the combining ability based heterotic grouping of inbred lines. The availability of low cost and high throughput single nucleotide polymorphic (SNP) markers have facilitated molecular characterization of a wide range of maize germplasm [12–15]. SNPs can be obtained using one of the various uniplex or multiplex genotyping and sequencing platforms that combine a variety of chemistry, detection methods and reaction formats [16]. Genotyping by sequencing (GBS) [17] has become one of the most popular methods in generating high density genome-wide SNP data relatively cheaply [2].

Several marker based studies were conducted to determine the genetic diversity, relationship, population structure, and heterotic grouping of CIMMYT [12–14, 18–21] and IITA [13, 15, 22, 23] maize inbred lines using different genotyping platforms and marker density. Some studies were also conducted to assess the genetic diversity and relationship of small number of maize inbred lines from the Ethiopian breeding program with small marker density [24–27]. Recently, we evaluated the level of genetic purity and identity among two to nine seed sources of 16 parental inbred lines of commercial hybrids using 191 Kompetitive Allele Specific PCR (KASP) and 257, 268 GBS markers [2]. Results from that study showed highly variable genetic purity that varied from 49 to 100%. Most of the inbred lines that originated from CIMMYT were highly homogenous whereas less than 25% of the inbred lines originated from EIAR’s breeding program were considered homogenous. These findings prompted us to assess the genetic purity of all inbred lines available to EIAR’s mid-altitude sub-humid maize breeding program. To the best of our knowledge, an extensive study on large number of maize inbred lines developed by three breeding institutions and widely used in Ethiopian maize breeding program has not been reported. The present study was, therefore, conducted to 1) assess the level of genetic purity among 265 maize inbred lines developed and/or widely used by breeders in the mid-altitude sub-humid maize agro-ecology of Ethiopia, 2) estimate the genetic distance and relatedness among the inbred lines, and identify redundant lines, and 3) assess the population structure and heterotic patterns of the inbred lines based on high density SNPs.

## Methods

### Plant materials and genotyping

A total of 265 inbred lines developed by EIAR, CIMMYT and IITA and widely used in the maize breeding program for the mid-altitude sub-humid maize agro-ecology of Ethiopia were used in this study (Additional file 1). Seed samples were obtained from the National Maize Research Coordinating Centre at Bako, Ethiopia. Based on their origin, the inbred lines are divided into three groups. One hundred and fifty of the inbred lines were developed and maintained by EIAR, hereafter referred to as ‘EIAR’. The second set, hereafter referred to as “CIMMYT”, consisted of 85 inbred lines developed by the CIMMYT’s global maize program, and the third set, hereafter referred to as “IITA”, consisted of 30 inbred lines developed by IITA. The inbred lines of CIMMYT and IITA were originally introduced from the two research institutes headquartered in Mexico and Nigeria, respectively, and are all currently being maintained at Bako Maize Research Station in Ethiopia. For each inbred line, genomic DNA was extracted from a bulked leaf sample collected from 10 seedlings using a modified version of CIMMYT’s high throughput mini-prep Cetyl Trimethyl Ammonium Bromide (CTAB) method [28]. DNA concentration was measured using the Quant-iT™ PicoGreen® dsDNA assay kit (Invitrogen™, Paisley, UK) and the Tecan Infinite F200 Pro Plate Reader (Grödig, Austria), and normalized to 50 ng μL^−1^. DNA quality was measured as described in our previous study [2] and shipped to the Institute of Biotechnology at Cornell University (http://www.biotech.cornell.edu/brc/genomics-facility). DNA samples of the 265 inbred lines in this study plus several other inbred lines with known pedigree and genetic purity from previous studies [2, 12] were genotyped with GBS at the Institute of Biotechnology, Cornell University, USA using *ApeKI* as restriction enzyme and 96-plex multiplexing as described by Elshire and colleagues [17].

### Data analyses

Imputed GBS data for a total of 955,120 SNP loci distributed across the ten maize chromosomes was received from the Institute of Biotechnology, Cornell University, USA. The genotype data was initially filtered using a minor allele frequency (MAF) of 0.05 and a minimum count of 80% of the sample size using TASSEL v.5.2.24 software [29], which gave 220,878 polymorphic SNPs, which is hereafter designated as “dataset 1” for further analyses (Table 1). The proportion of heterogeneity (the number of markers that were not homozygous due to mixture of two or more homozygous genotypes due to bulking or residual heterozygosity) in each inbred line was calculated from dataset 1 using TASSEL v5.2.24. Genetic purity was calculated in Excel as the difference between 100-h, where h refers to heterogeneity in percentage obtained from TASSEL. Genetic distance was calculated between pair of inbred lines in dataset 1 using the identity by state (IBS) method implemented in TASSELv.5.2.24. A relative kinship matrix was calculated between pair of inbred lines in dataset 1 using TASSELv.5.2.24, while a kinship heatmap was computed from the same dataset using the R package for Windows-based Genome Association and Prediction Integrated Tool (GAPIT) v.2.0 [30]. Principal coordinate analysis was performed from the genetic distance matrix using the Dissimilarity Analysis and Representation for windows (DARwin) v.6.0.013 (http://darwin.cirad.fr).

**Table 1:** The chromosomal distribution and proportion of polymorphic markers used for computing heterogeneity, genetic distance, relative kinship, and principal coordinate analyses (dataset 1), and population structure and DAPC (dataset 2)

Chromosome	Dataset 1		Dataset 2	
	No. of markers	Proportion	No. of markers	Proportion
1	35,002	16%	3391	16%
2	26,547	12%	2539	12%
3	25,311	11%	2347	11%
4	20,748	9%	2353	11%
5	26,051	12%	2358	11%
6	17,822	8%	1696	8%
7	18,630	8%	1823	8%
8	18,763	8%	1941	9%
9	16,680	8%	1658	8%
10	15,324	7%	1539	7%
Total	220,878	100%	21,645	100

Population structure was estimated from 21,645 SNPs, hereafter designated as “dataset 2” obtained after further filtering the 220,878 polymorphic SNPs using a MAF of 0.10 and a physical distance of 10 kb between adjacent markers. Additional filtering was done to select evenly distributed SNPs across the ten maize chromosomes that can easily be handled by the software used for population structure analysis. Population structure was estimated using both an admixture model-based clustering method implemented in the software package STRUCTURE v.2.3.4 [31] and the Discriminant Analysis of Principal Components (DAPC) [32] based on the adegenet package implemented in the R for Windows v64.3.3.2. For DAPC, the best number of groups was identified using the “*find.clusers”* function in R, which runs K-means with increasing values of K from 1 to 12. We compared the different groups using Bayesian Information Criterion (BIC), with the lowest BIC value used to infer the ideal number of groups (sub-populations). The first two principal components from DAPC were plotted for visual examination of the clustering pattern of the inbred lines. The model-based clustering in STRUCTURE v.2.3.4 was ran by varying the number of clusters (K) from 1 to 12, with each K repeated thrice with a burn-in period of 10,000 and 10,000 MCMC (Markov Chain Monte Carlo) replications after burn-in. Individuals with probability of membership ≥60% were assigned to the same group, while those with <60% probability memberships in any group were assigned to a “mixed” group [12, 15].

## Results and discussion

### Markers and genetic purity

**Fig. 1:**
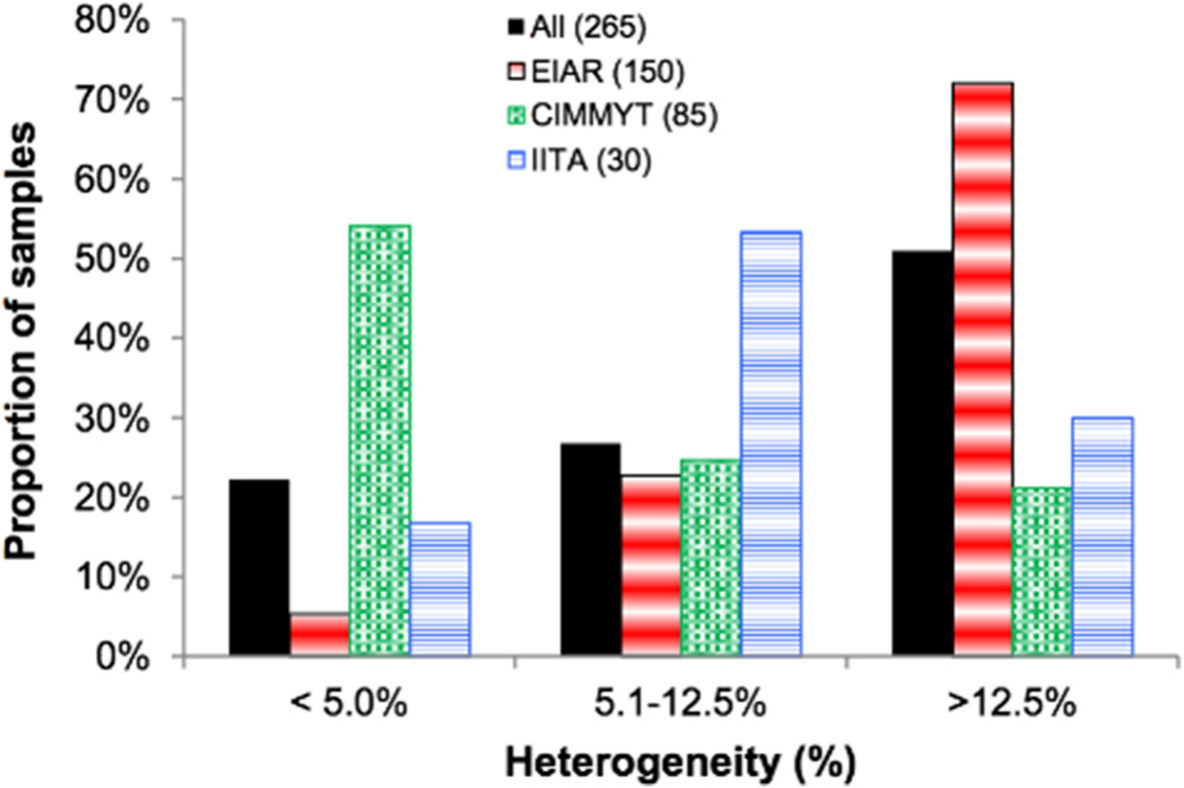
Summary of the heterogeneity of 265 inbred lines based on 220,878 polymorphic SNPs. The number of inbred lines are shown in brackets in the legend

Among the 955,120 SNPs used for genotyping the 265 inbred lines, only 23.1% (220,878 SNPs) were polymorphic in our germplasm, each with a minor allele frequency ranging from 0.05 to 0.50. The percentage of missing data per marker after imputation varied from 0 to 19.2% and the overall average was 9.2%. The number of SNPs per chromosome for dataset 1 varied from 15,324 SNPs on chromosome 10 to 35,002 SNPs on chromosome 1 (Table 1). Using 220,787 SNPs, genetic purity among the 265 inbred lines varied from 68.5 to 99.9% (Additional file 1), with an overall average of 86.9%. Genetic purity of inbred lines is an important quality control criteria in maize breeding and seed system, that directly affects both the quality of hybrid seed and development of new inbred lines [2, 33]. Currently, most maize breeding programs consider S_4_ or later generation as a fixed inbred line for evaluation in hybrid combination. Inbred lines are considered pure or fixed when the proportion of heterozygous SNP loci does not exceed 5% [33]. Inbred lines with higher than 5% heterogeneous SNP loci are considered either not fixed or likely to have been contaminated by pollen or seed of another source during maintenance. Overall, about 22% of the 265 inbred lines were considered fixed, while the remaining 27% and 51% of the inbred lines had a heterogeneity varying from 5.1 to 12.4 and from 12.5 to 31.5%, respectively (Fig. 1, Additional file 1). Approximately 7% of EIAR’s, 20% of IITA’s and 54% of CIMMYT’s inbred lines were considered fixed. Most inbred lines from EIAR (73%) showed heterogeneity values ranging from 12.5 to 31.5% as compared to only 21% from CIMMYT and 30% from IITA (Fig. 1, Additional file 1). The higher level of heterogeneity observed for most inbred lines from EIAR was due to the use of early generation inbred lines (<S_4_) as parents for hybrid formation. This approach was used to attain higher seed yield in the prevailing poor inputs and agronomic practice. This in turn lowers the price of hybrid seed production thereby decreasing cost of seed and increasing access to seed by small scale farmers [34]. In addition, the source germplasm available for new line development some decades ago (composites, pools and landraces) showed sever inbreeding depression upon continuous self-pollination. To cope with those challenges, maize breeders in Ethiopia at that time developed and released hybrids using early generation parental inbred lines [3]. Although this strategy favored cheaper seed production, the hybrids were less uniform in comparison to hybrids developed from fixed lines. Also, the genetic purity of some of the recently developed EIAR inbred lines was low, possibly due to pollen contamination and seed admixture during seed maintenance. The majority of the inbred lines originating from both CIMMYT and IITA were at S_4_ or later generations [33], and thus, both pollen contamination and seed admixture during inbred line maintenance could be the most likely factor that resulted in higher level of heterogeneity in some of these lines. We therefore suggest an additional generation of selfing in order to fix these inbred lines (with the exception of those deliberately maintained at an early stage) to achieve a number of advantages from the use of pure lines, including ease of maintenance of parental lines, high heterosis in hybrids, and ease in quality control during hybrid seed production [2, 33]. We also suggest periodic restocking of inbred lines sourced from IITA and CIMMYT as well as maintenance of reference molecular fingerprints for ease of identity confirmation in future and internal quality control. Further, it is recommended to complement marker based homogeneity test with phenotypic evaluation at regular interval (e.g., every five years) to verify the genetic purity of inbred lines.

One of the limitations of GBS markers was concerns on the reliability of allele calls on heterogeneous and highly heterozygous germplasm as compared with highly homozygous genotypes, which has been dealt through intensive post data correction, including implementation of reliable imputation methods [35, 36]. Using 191 SNPs from Kompetitive Allele Specific PCR (KASP) and different number of GBS markers, we recently compared genetic purity of 80 maize samples (16 maize inbred lines, each represented from 2 to 9 seed sources). The KASP and GBS-based SNP markers showed some discrepancy in terms of numerical values when heterogeneity exceeded 12.5%, but the overall conclusions reached in assigning lines into genetically pure or not were highly similar. The correlation between KASP and GBS markers for estimating genetic purity varied from 0.90 to 0.93 depending on the number of GBS markers used for analyses [2]. The KASP-based SNPs are preselected high quality SNPs for QC analysis but they are much fewer than the number of GBS markers used for estimating genetic purity, which may be one of the reasons for the observed small differences between KASP and GBS.

### Genetic distance and kinship

**Fig. 2:**
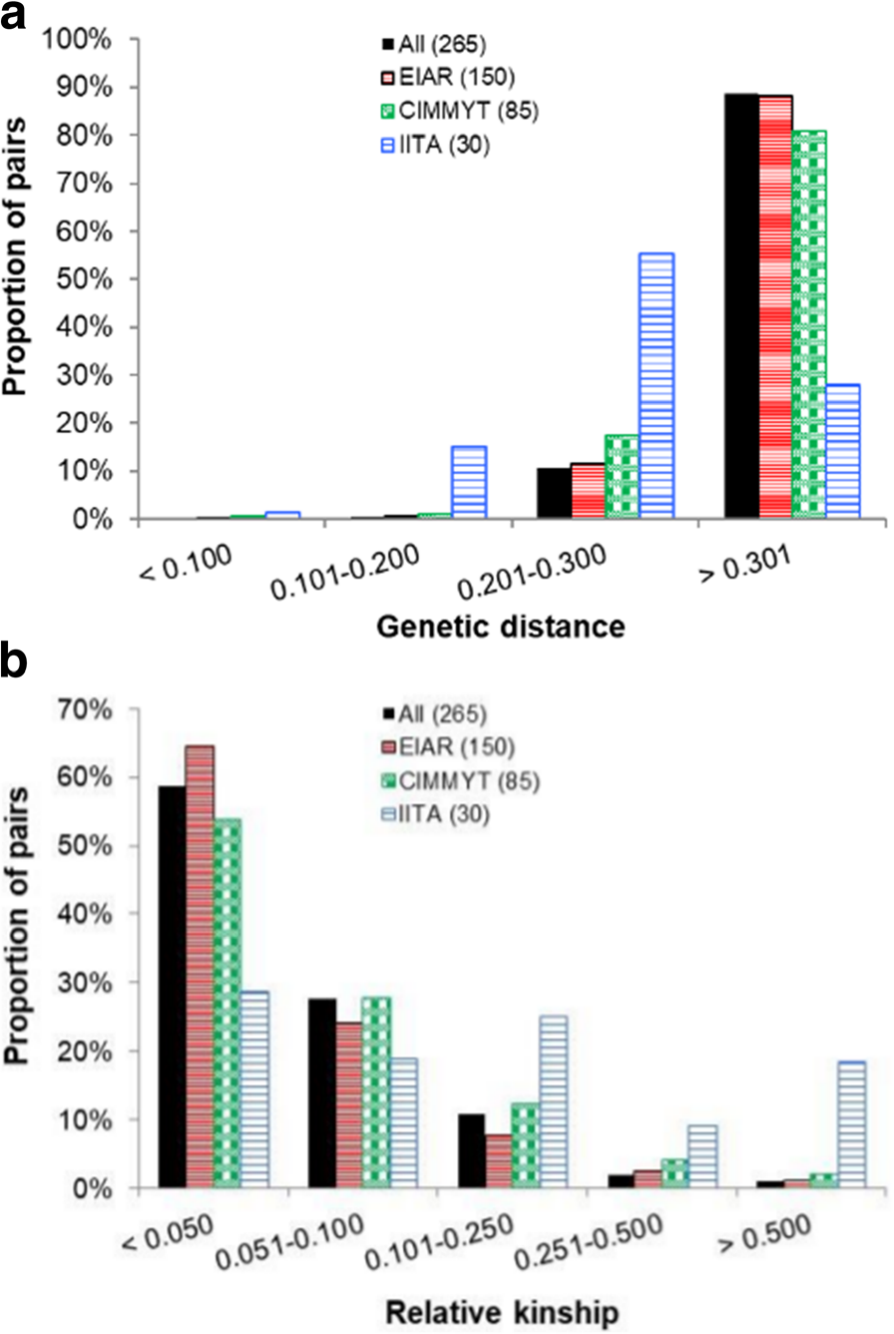
Summary of (**a**) genetic distances and (**b**) kinship coefficients between pair of inbred lines from different sources based on 220,878 polymorphic SNPs. The number of inbred lines are shown in brackets in the legend

Pairwise genetic distances among the 265 inbred lines ranged from 0.011 to 0.346 (Additional file 2), with an average of 0.313. Only fourteen pairs (0.04%) of inbred lines showed genetic distance estimates less than 0.05 with most of these pairs originating from CIMMYT. All pairs of the inbred lines with a genetic distance <0.05 were sister inbred lines, with shared pedigree across most generations. The proportion of pairwise comparisons with a genetic distance estimate less than 0.200 was ≤1% for inbred lines originating from EIAR and CIMMYT and was 15% for inbred lines originating from IITA (Fig. 2a). The genetic distance between pairs of inbred lines in 89% of the entire set, 88% of EIAR and 81% of CIMMYT fell in the range of 0.301 to 0.346 (Fig. 2a). Most IITA inbred lines had a genetic distance estimate between 0.200 and 0.300 (55%), while only 28% of them had genetic distance estimate between 0.301 and 0.346. The result suggested relatively narrow genetic variation among the sampled inbred lines of IITA compared to those of EIAR and CIMMYT. This could be due to differences in sample sizes of the lines included in the present study from the different institutes and the heterotic patterns of the lines as defined in the various programs. Previous genetic distance estimates reported for tropical maize germplasm are highly variable. In one of the recent studies, approximately 59% of the pairwise distances among 417 doubled haploid maize lines genotyped with 97,190 GBS markers ranged between 0.301 and 0.500 [37]. In another study involving 450 inbred lines developed by CIMMYT breeders in Africa, 95% of the pairs of inbred lines showed genetic distance values ranging between 0.301 and 0.500 [12].

Selection of parents with good phenotypic performance and wide genetic base is one of the most important steps in the development of new hybrid varieties. In general, progeny variance increases in crosses between genetically distant parents [38], providing opportunities to generate progenies with maximum segregation for target traits that are desired. Breeders use different methods in selecting the best parents for making new crosses, including (a) pedigree relationships, (b) phenotypic performance for specific traits, (c) adaptability and yield stability, and (d) genetic distances estimated from phenotypic traits and molecular markers [39]. The relationship between genetic distance and progeny genetic variance was found to be inconsistent across species and studies, with some showing strong relationships, while others showing weak relationships [8, 9, 39–41]. Nevertheless, genetic distances estimated from high density molecular markers could provide useful additional information for selecting the best parental combination to generate new crosses for developing improved maize inbred lines.

The pairwise relative kinship coefficients among the 265 inbred lines ranged between 0.00 and 1.778, where values close to zero indicate lack of relationship, while those close to 2 indicate complete relationship. Fifty-nine percent of the relative kinship values were close to zero, 40% varied between 0.050 and 0.500, and the remaining 1% fell between 0.500 and 1.778 (Fig. 2b, Additional file 3). As shown in Additional file 4, the kinship heatmap computed using the 220,878 SNPs reached a pick between zero and 0.050, which shows lack of relatedness in most pairs of inbred lines used in this study. The proportion of close to zero pairwise kinship values observed in the presents study (59%) was much higher than the one reported for the 450 inbred lines originating from the CIMMYT Africa maize breeding program which was only 5.1% [12], and was relatively higher than that of the 632 inbred lines reported for the global maize collection [18]. Similar results of close to zero pairwise relative kinship values were reported in 61% of the 100 inbred lines from INERA and IITA [22], 60% pairs of 359 inbred lines from CIMMYT and IITA [13], and 64% pairs of 544 inbred lines from CIMMYT [14]. Considering relative kinships within each of the three germplasm sources, EIAR depicted the highest percentage (64%) of pairs of lines with kinship values close to zero, followed by CIMMYT (54%), and IITA (29%) (Fig. 2b). Assemblage of maize germplasm from diverse sources might have contributed to the observed low level of relatedness among EIAR’s inbred lines.

### Genetic relationship and population structure

**Fig. 3:**
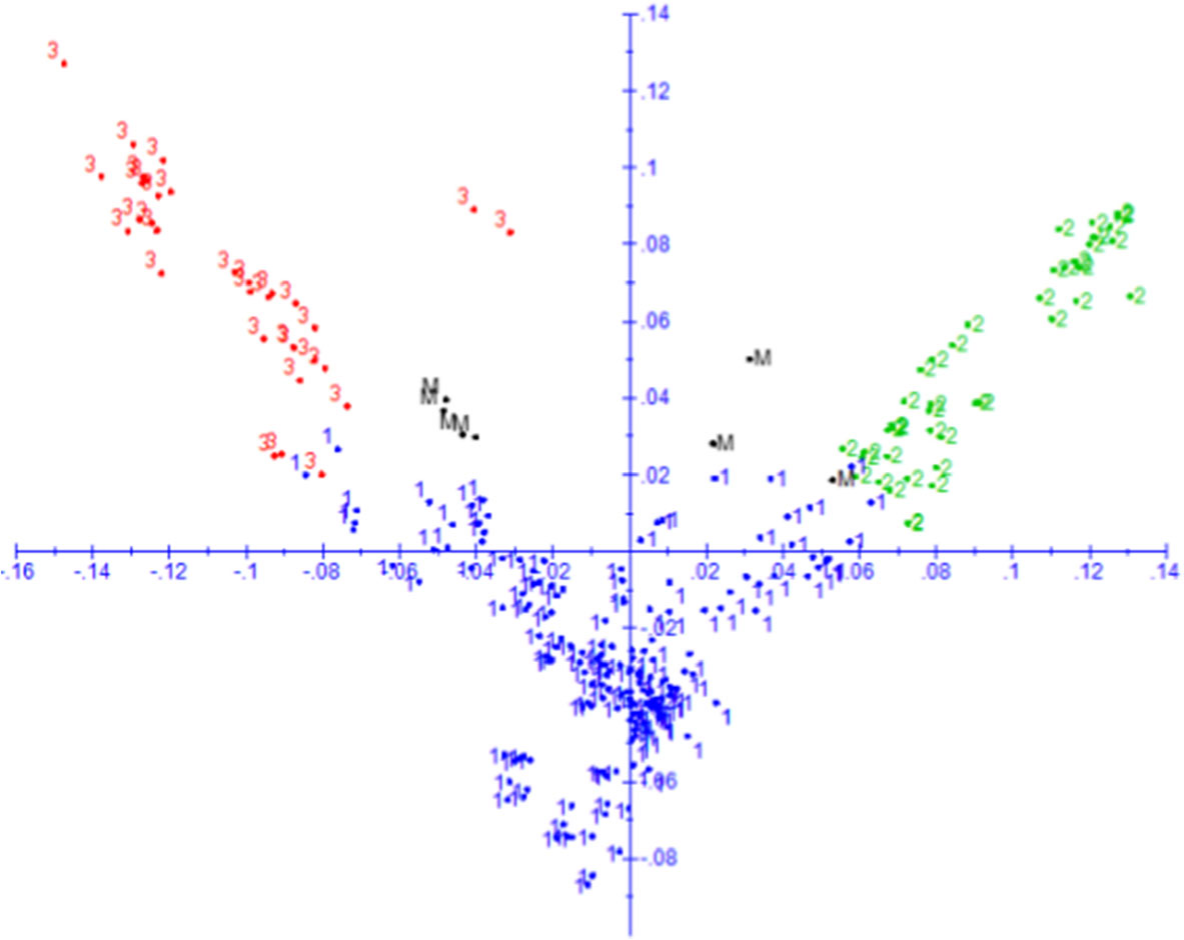
Plot of PC1 (7.1%) and PC2 (4.5%) from principal coordinate (PC) analysis of 265 inbred lines based on genetic distance matrix calculated from 220,878 SNPs. The plot was made using predicted group membership from STRUCTURE (group 1 = blue; group 2 = green; group 3 = red; mixed group = M, black). See Additional file 1 for details on membership of each group

**Fig. 4:**
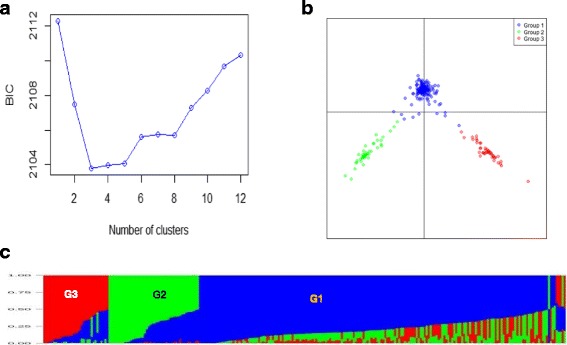
Population structure of 265 inbred lines based on 21,645 SNPs using Discriminant Analysis of Principal Components (DAPC) and the model-based STRUCTURE: **a** Bayesian Information Criterion (BIC) values showing number of possible clusters ranging from 1 to 12; **b** Plot of PC1 and PC2 from DAPC showing the three groups; (**c**) groups predicted based on STRUCTURE at K = 3 (group 1 = G1; group 2 = G2, and group 3 = G3). In STRUCTURE, each inbred line is represented by a thin vertical bar, which is partitioned into three colored segments (blue = G1, green = G2 and red = G3) on the x-axis, with lengths proportional to the estimated probability membership on the y-axis

The population structure of the inbred lines was assessed using PCA, DAPC and the model-based STRUCTURE. All the three methods revealed the presence of three distinct groups, with 94% agreement on group membership predicted by the different methods (Figs. 3-4 and Additional file 1). Using DAPC, the first group was composed of 175 quality protein maize (QPM) and non-QPM inbred lines that were mainly extracted from broad-based pools and populations, such as PooL9A for non-QPM lines and Pop 62 and Pop 63 for QPM inbred lines. Pool 9A, which is considered as heterotic group A (HGA) population, was developed from a pool of Kitale synthetic II (HGA), Ecuador 573 ((heterotic group B (HGB), Colombian, Guatemalan, Tuxpeño (HGA) and SR52 (HGA/HGB) [42, 43]. Pool 9A is adapted to the highland transition-zone growing conditions and characterized by late maturity, semi-dent texture, and white grain. Pop 62, on the other hand, was originally derived from pool 40 [44]. Like Ecuador 573, pool 40 was developed for both the intermediate temperate ranges and colder maize growing areas of the tropics and subtropics [45]. Other germplasm sources included in this group also include pop 43, INTB, DRB, ZM605, ZM609, EV7992 and TZM, which represent both HGA and HGB germplasm. Some popular CIMMYT HGA testers (CML312 and CML442) and HGB testers (CML444) were also clustered in group one, highlighting the discrepancy between marker based and combining ability based heterotic groupings.

Group two consisted of 47 members, including CML395, CML202 and several other inbred lines recycled from CML395 and/or CML202, whereas group three consisted of 43 inbred lines that were primarily derived either from Ecuador 573 or CML197 genetic backgrounds. CML395 and CML202 are popular CIMMYT HGB testers used in tropical mid-elevation adapted germplasm in SSA as both carry resistance to maize streak virus (MSV). Ecuador 573, which is an OPV originally obtained from Ecuador and improved through reciprocal recurrent selection with Kitale synthetic II [3, 43], is a popular HGB population adapted to highland growing conditions and characterized by late maturity and flint kernel texture. Ecuador 573 and Kitale synthetic II and inbred lines extracted from them have been extensively used as parents and testers in developing improved germplasm adapted to upper mid-altitude sub-humid and transitional highland sub-humid maize growing areas of Ethiopia [3]. If we rely only on pedigree information, inbred lines in groups two and three should belong to the same heterotic group (HGB). However, the magnitude of genetic distance and heterosis supports the molecular marker grouping. The highest genetic distance between pairs of lines in the present study (0.346) was between CML395 from group two and 142–1-e (derived from Ecuador 573) from group three. Furthermore, BH661, a high yielding three-way cross hybrid released in Ethiopia in 2011 [11] is a cross between a single cross hybrid from group two (CML395/CML202) and 142–1-e. This clearly supports the population structure detected between group two and three in the present study. Therefore, the population structure defined by the molecular data appears more plausible than the conventional heterotic grouping based on pedigree and combining ability studies that clusters CML395 and EC 573 into the same group.

**Fig. 5:**
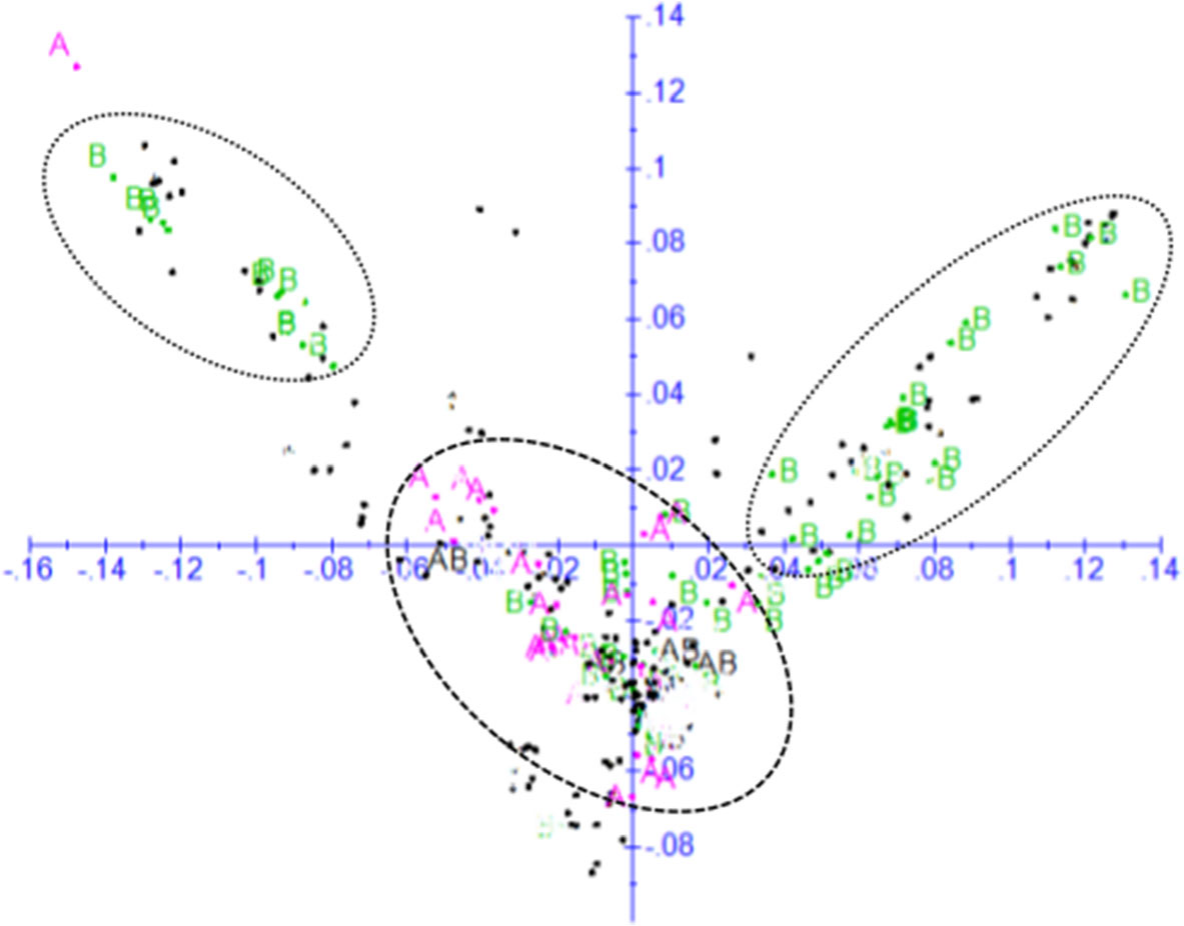
Plot of PC1 (7.1%) and PC2 (4.5%) from principal coordinate (PC) analysis based on genetic distance matrix calculated from 220,878 SNPs. Only inbred lines that belongs to heterotic group A and B are shown in pink and green font, respectively. Note that inbred lines in heterotic group B are primarily divided in two subgroups, while those in heterotic group A belong to a single group (see Additional file 1 for details)

As shown in Fig. 5, results from the present study showed partial agreement with the conventional method of heterotic group designation in tropical inbred lines, but the pattern was not very distinct. Most HGA inbred lines clustered into group 1, while HGB inbred lines were found distributed across all the three groups; most, however, were in the second and third groups. Our findings on the lack of clear pattern of grouping based on the germplasm origin, and inconsistency between molecular marker-based clustering and the conventional classification is in agreement with previous studies on tropical maize germplasm [12, 14, 22] that reported inconsistencies between molecular marker-based and combining ability/pedigree based classifications. As the assignment of inbred lines in to heterotic groups in tropical maize germplasm is a relatively recent phenomenon, the lack of clear genetic divergence between HGA and HGB lines in the current study is not entirely unexpected. Until the early 1990s, most of the tropical maize germplasm improvement effort of CIMMYT, IITA and most national agricultural systems (NARS) was based on the development of pools and populations with stacked traits with the objective of deriving adapted OPVs without consideration to heterotic pattern. With the advent of the seed sector and maize hybrid adoption in the tropics, the focus of CIMMYT and IITA switched to inbred line development in the early 1990s and subsequent assignment of germplasm into distinct heterotic groups. Most tropical germplasm was assigned to either HGA (Tuxpeno, flint background) or HGB (ETO (Estacion Tulio Ospina), dent) which were found to combine well with each other while a small fraction of germplasm was assigned to heterotic group AB (HGAB) due to lack of a clear combining pattern. Due to the high levels of diversity in tropical maize germplasm, it is likely to take several decades before HG can reliably be identified by molecular marker, phenotype or combining ability. The current assignment of heterotic groups to inbred lines is based on test cross performance with various representative testers. However, it remains challenging to divide tropical maize inbred lines into clear heterotic groups based on combining ability results per se as many of them are derived from mixed pools while selection within each heterotic group has not been carried out for long enough to achieve maximum heterotic response between groups [19]. Therefore, many generations of reciprocal recurrent selection may be needed before inbred lines from each heterotic group begin to be significantly divergent [21]. In addition, combining ability based heterotic group assignment relies on yield performance evaluation of different sets of lines with different testers. The reliability of combining ability based heterotic grouping depends on several factors, including (1) the genetic background of the inbred lines, (2) the type and number of testers used, (3) inconsistencies in the number of environments used for yield trials, (4) the involvement of different breeders from the same or different institutions and use of different testers; and (5) lack of common check hybrids across different yield trials that could be used for comparing results across institutions, breeders, and years. Given such limitations on the combining ability based heterotic grouping, the partial agreement between the phenotypic and molecular-based heterotic grouping is expected. Hence, our results, together with others [12, 14, 15] suggest the need for complementing combining ability based assessment with a molecular fingerprinting and pedigree history when determining heterotic groups.

Lack of clear grouping of the inbred lines in this study based on their origin (EIAR, CIMMYT or IITA) is partly attributed to EIAR’s continuous acquisition of germplasm from both CIMMYT and IITA, and also germplasm exchange among maize breeders at CIMMYT and IITA. Maize breeders from the NARS in SSA often have limited source germplasm for their breeding program and are mainly dependent on CIMMYT and /or IITA maize germplasm. CIMMYT provide free access to over 576 publicly available CMLs and many other advanced inbred lines to maize breeder’s worldwide. Adapted inbred lines received from CIMMYT and IITA are crossed to various locally developed maize inbred lines to derive new improved inbred lines and hybrids. The classification of CIMMYT/IITA inbred lines into three heterotic groups (A, B or AB), has indirectly influenced many of the NARS breeders in SSA to adopt a similar system of heterotic grouping, which is essential for establishing a line and hybrid development pipeline.

## Conclusions

Genetic variation and population structure of maize inbred lines available for the mid-altitude sub-humid agro-ecology in the maize breeding program of Ethiopia were assessed using high density SNP markers. Results of the study demonstrated the presence of high level of heterogeneity within most of the inbred lines studied, suggesting the requirement for additional generation of inbreeding to obtain fixed inbred lines, as deemed necessary. Genetic distances among most pairs of inbred lines clearly showed the high level of variation among the most inbred lines available for the mid-altitude sub-humid maize agro-ecology of Ethiopia. Although many of the inbred lines developed by EIAR shared the genetic background of some CIMMYT’s and IITA’s popular inbred lines, a high level of genetic variation was found among most EIAR inbred lines. The different multivariate methods revealed the presence of three distinct groups that is broadly in agreement with the genetic backgrounds of the inbred lines. There was no clear pattern of grouping of the inbred lines along institutional origin (EIAR, CIMMYT and IITA), which could be due to the continuous exchange of genetic material and development of many inbred lines using the CIMMYT and IITA inbred lines as parents. Our results provide useful information about the extent of genetic variation and population structure of maize inbred lines developed and/or widely used by breeders in the mid-altitude sub-humid maize agro-ecology of Ethiopia. The result also facilitates germplasm exchange among breeding programs in SSA.

## Additional files

Additional file 1: Summary of the 265 inbred lines and population structure. (XLSX 47 kb)

Additional file 2: Genetic distances between pairs of 265 inbred lines based on 220,878 polymorphic SNPs. (XLSX 447 kb)

Additional file 3: Relative kinship coefficients between pairs of 265 inbred lines based on 220,878 polymorphic SNPs. (XLSX 507 kb)

Additional file 4: Heat map of the 265 inbred lines based on relative kinship matrix estimated from 220,878 polymorphic SNPs. (DOCX 211 kb)

## Abbreviations

CIMMYT: International maize and wheat improvement center
CTAB: Cetyl trimethyl ammonium bromide
EIAR: Ethiopian institute of agricultural research
GAPIT: Genome association and prediction integrated tool
GBS: Genotyping-by-sequencing
HGA: Heterotic group A
HGAB: Heterotic group A and B
HGB: Heterotic group B
IBS: Identity by state
IITA: International institute of tropical agriculture
KASP: Kompetitive allele specific PCR
MAF: Minor allele frequency
NARS: National agricultural research systems
OPVs: Open-pollinated varieties
QC: Quality control analysis
SNP: Single nucleotide polymorphism
SSA: Sub Saharan Africa
