# Supplementary material for: Genetic variation and population structure of maize inbred lines adapted to the mid-altitude sub-humid maize agro-ecology of Ethiopia using single nucleotide polymorphic (SNP) markers
Source: BMC Genomics. 2017 Oct 12;18:777. doi: 10.1186/s12864-017-4173-9 (PMC5639748; doi:10.1186/s12864-017-4173-9)
Supplement: Supplementary file 4 — Heat map of the 265 inbred lines based on relative kinship matrix estimated from 220,878 polymorphic SNPs. (DOCX 211 kb) [file 12864_2017_4173_MOESM4_ESM.docx]

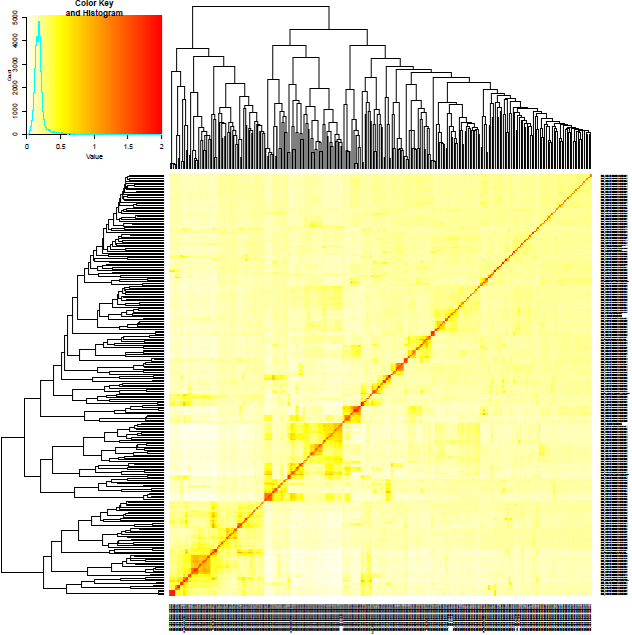


Additional file 4: Heat map among pairs of the 265 inbred lines based on relative kinship matrix estimated from 220,878 polymorphic SNPs.
